# The effect of a comprehensive typhoid conjugate vaccine campaign on antimicrobial prescribing in children in Harare, Zimbabwe: a mixed methods study

**DOI:** 10.1016/S2214-109X(23)00319-4

**Published:** 2023-09-01

**Authors:** Ioana D Olaru, Rudo M S Chingono, Christian Bottomley, Faith R Kandiye, Fadzaishe Mhino, Chipo A Nyamayaro, Salome Manyau, Michael Vere, Phillomina Chitando, Prosper Chonzi, Thomas C Darton, Justin Dixon, Katharina Kranzer

**Affiliations:** Biomedical Research and Training Institute, Harare, Zimbabwe; Clinical Research Department; Institute of Medical Microbiology, University Hospital Münster, Münster, Germany; Biomedical Research and Training Institute, Harare, Zimbabwe; Department of Infectious Disease Epidemiology; Biomedical Research and Training Institute, Harare, Zimbabwe; Biomedical Research and Training Institute, Harare, Zimbabwe; Department of Global Health and Development London School of Hygiene & Tropical Medicine, London, UK; Department of Health, Harare City Council, Rowan Martin Building, Harare, Zimbabwe; Department of Infection, Immunity and Cardiovascular Disease, University of Sheffield, Sheffield, UK; Biomedical Research and Training Institute, Harare, Zimbabwe; Department of Global Health and Development London School of Hygiene & Tropical Medicine, London, UK; Biomedical Research and Training Institute, Harare, Zimbabwe; Clinical Research Department; Division of Infectious and Tropical Medicine, Medical Centre of the University of Munich, Munich, Germany

## Abstract

**Background:**

Vaccines prevent infections and could subsequently reduce antimicrobial use. A 1-week mass vaccination campaign was done with Typbar-TCV (Bharat Biotech, Hyderabad, India) between Feb 25 and March 4, 2019. We investigated whether this typhoid conjugate vaccine campaign could affect antimicrobial prescribing in children presenting to primary care in Harare, Zimbabwe.

**Methods:**

In this mixed methods study, data for acute paediatric outpatient consultations between Jan 1, 2018, and March 31, 2020, were collected from five clinics in Harare. Interrupted time series analysis was done to compare prescription data before and after the campaign. To contextualise findings, qualitative data were collected between April 20, 2021, and July 20, 2022, comprising ethnographic research (ie, workshops, surveys, observations, and interviews) in 14 clinics. Ethnographic data were used for thematic analysis. The primary outcome was monthly antimicrobial prescriptions in children aged 6 months to 15 years, normalised by the number of trauma events in all age groups.

**Findings:**

In the data collection period, 27 107 paediatric consultations were recorded. 17 951 (66·2%) of 27 107 children were prescribed antimicrobials. Despite the perceived reduction in typhoid cases and a decreasing trend in the prescription of antimicrobials commonly used to treat typhoid (ie, ciprofloxacin and azithromycin), mass vaccination with Typbar-TCV did not affect the total rate of antimicrobials (adjusted rate ratio, 1·20, 95% CI 0·70–2·05, p=0·51) or the rate of typhoid antimicrobials prescribed (0·93, 0·44–1·96, p=0·85). Unsafe water sources and insufficient diagnostic services were reported to contribute to the continued disease burden and antimicrobial prescription.

**Interpretation:**

Non-specific febrile illness caused by confirmed or suspected typhoid is a common cause of antimicrobial use in endemic areas. Although effective in preventing typhoid fever, we were unable to identify any effect of Typbar-TCV on antimicrobial prescribing. Ethnographic research showed the effect of contextual factors on antimicrobial prescribing, including concerns regarding safe water access, appropriate sewage disposal, health-care and diagnostic availability. To realise effects beyond disease burden reduction, holistic approaches addressing these concerns are needed so that the value of vaccines mitigating the effects of antimicrobial use as a driver of antimicrobial resistance is fully achieved.

**Funding:**

Wellcome Trust.

## Introduction

Globally an estimated 14·3 million cases of enteric fever (ie, typhoid and paratyphoid) occurred in 2017, leading to considerable morbidity and antimicrobial use.^1^ Low availability of blood culture facilities and insufficient accuracy of non-culture tests makes diagnosis in low-resource settings challenging.^2,3^ The decision to prescribe antimicrobial treatment is often based on clinician experience or a syndromic approach.^4^ In south Asia, undifferentiated febrile illnesses including typhoid are thought to be key drivers of antimicrobial use, which might in turn drive antimicrobial resistance.^5^ However, only one patient in a group of between four and 26 patients treated for suspected typhoid fever have a confirmed infection with *Salmonella enterica* serotype Typhi (*S* Typhi), depending on the population and setting.^5^ Increasing reports of typhoid fever as a cause of undifferentiated febrile illness in sub-Saharan Africa are raising similar concerns regarding antimicrobial stewardship and the effect on antimicrobial resistance in this region.^6^

Harare, the capital of Zimbabwe, has had recurrent outbreaks of typhoid fever and cholera over the past decade.^7,8^ During an outbreak in 2018–19, 19% of individuals presenting with fever to a primary care clinic in Harare had a blood culture positive for *S* Typhi and decreased susceptibility to fluoroquinolones was common.^9^ In this setting, routine diagnostic testing for typhoid is not available, resulting in frequent empirical use of antimicrobials for suspected cases.

Vaccines could be a promising tool for mitigating the growing threat of antimicrobial resistance,^10,11^ and their use for this purpose is supported by WHO.^12,13^ In December, 2017, a new typhoid conjugate vaccine (Typbar-TCV, Bharat Biotech, Hyderabad, India) received WHO prequalification for use in individuals aged at least 6 months.^14^ WHO recommended that Typbar-TCV should be prioritised for outbreak control and in settings with high prevalence of antimicrobial resistance.^15^ This instance is the first in which WHO has recommended a vaccine based on pathogen-specific antimicrobial resistance concerns. To control the ongoing typhoid outbreak in Harare, a 1-week mass vaccination campaign was done, using the Typbar-TCV vaccine in the area for the first time (Feb 25 to March 4, 2019). All children aged 6 months to 15 years living in the nine densely populated suburbs with the greatest typhoid burden were targeted for vaccination,^16^ and vaccine coverage reached 88% in these selected areas.^17^ Half of the Harare population lives in suburbs where vaccination was done.^18^ A combination of strategies were used to reach the target population (fixed and mobile sites, vaccination in schools, and door-to-door visits).^17^ As expected, vaccination had a substantial effect in reducing the incidence of confirmed typhoid cases in the vaccinated populations.^9,19^

In this study we investigate the effect of vaccination on antimicrobial prescribing in children presenting to primary care. We hypothesised that, given the high burden of typhoid fever in this setting, the Typbar-TCV mass campaign would lead to a reduction in infections, which might result in a decrease in antimicrobial prescriptions overall and in antimicrobials used for typhoid fever. We also sought to understand the local factors influencing antimicrobial prescribing from the perspective of health-care workers and other stakeholders to provide an in-depth contextual appreciation of any changes following Typbar-TCV roll-out.

## Methods

### Study design

In this mixed methods study, we collected data for quantitative analysis from five public primary care clinics located in five of the nine vaccinated suburbs in southwest Harare (appendix 2 p 2). Qualitative data collection was done in the same five clinics and an additional nine clinics located in both vaccinated and unvaccinated suburbs. The additional clinics were included to gain a more representative understanding of typhoid, antimicrobial prescribing, and where and why any changes occurred across Harare.

### Procedures

Patient consultation data were extracted from paper-based outpatient clinic registers, which also contained sex data. Consultations done between Jan 1, 2018 (14 months pre-vaccination), and March 31, 2020 (12 months post-vaccination), were analysed. Data for the quantitative analysis were censored on March 31, 2020, when a strict COVID-19 lockdown led to health service disruption and clinic closures. All clinic presentations were analysed. Patients might have sought care multiple times during the study.

Ethnographic data were collected between April 20, 2021, and July 20, 2022, 2 years after the vaccination campaign. The longer post-vaccination period enabled us to capture the long-term effect, sustainability, and co-benefits of any changes in antimicrobial prescribing practices, but equally any factors as to why the vaccine might not have substantially influenced prescribing in a setting that has regular health system shocks and economic turmoil. We also used the FIEBRE study of antimicrobial prescribing in Harare to verify, interpret, and contextualise findings.^20^

The study was approved by the London School of Hygiene & Tropical Medicine (reference numbers 16424 and 21686) and Medical Research Council of Zimbabwe (MRCZ/A/2406 and MRCZ/A/2619) ethics committees. All participants in the qualitative component of the study provided written informed consent to take part. Consent was not required for collection of register data as no data were collected from individuals.

Data collection consisted of key event mapping, clinic surveys, participant observations, and a collaborative workshop (appendix 2 pp 4, 7–8) led by two research assistants (FRK, CAN) with the support of a postdoctoral social scientist (RMSC). Key event mapping involved a participatory workshop with 19 Harare City Health officials on June 12, 2021, to capture typhoid-relevant events in Harare between Jan 1, 2016, and Dec 31, 2021. A clinic survey was done in 14 clinics at two timepoints, at the start (April 20 to May 20, 2021) and end of data collection (May 8 to June 15, 2022) to capture community-specific events not discernible at the city level and gain a formative understanding of clinic routines and typhoid management.

**Figure 1:**
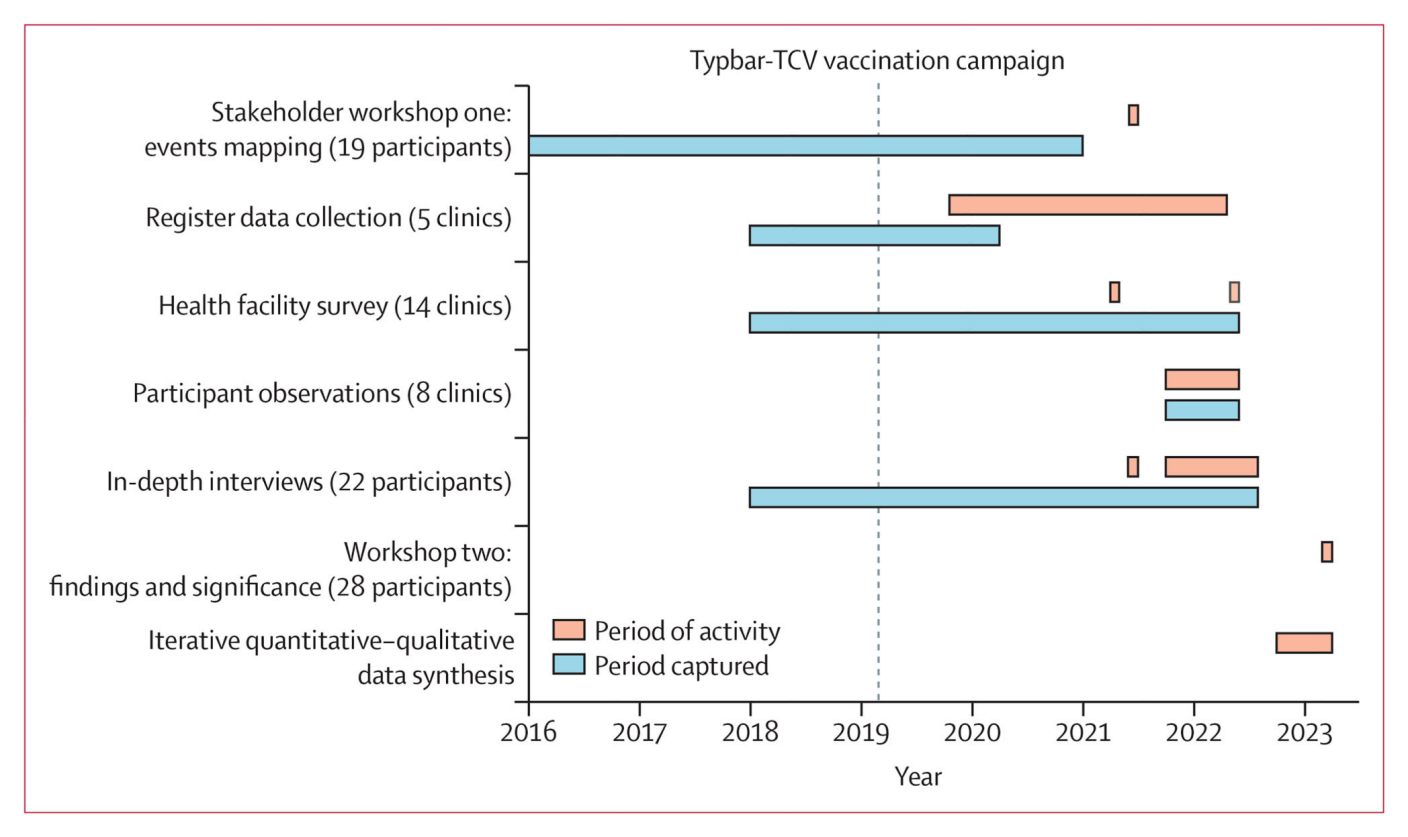
Timeline of study activities The timeline refers to the data presented in this manuscript.

Following analysis of the clinic survey in 2021, participant observation was done over 8 months (Oct 19, 2021, to May 5, 2022) in a smaller subset of eight clinics with 26 prescribing nurses and environmental technicians. Participant observation was designed to provide a granular understanding of typhoid prevention, diagnosis, and treatment; the factors taken into consideration (including vaccination status); and any challenges of following the Essential Medicines List and Standard Treatment Guidelines for Zimbabwe (EDLIZ). In-depth interviews were done for the periods of June 8 to July 20, 2021, and Oct 27, 2021, to July 20, 2022, with 22 prescribers, clinic pharmacy dispensary attendants, environmental health technicians, and clinic managers to provide context on findings from participant observation and perceptions around typhoid, Typbar-TCV, and their effects on prescribing practices. Interviews were audio recorded and transcribed. Finally, a second collaborative workshop was held on April 20, 2023 with 28 employees of Harare City Health Authorities to share and draw out the significance of study findings (figure 1).

### Statistical analysis

Interrupted time series analysis was done to compare antimicrobial prescribing before and after Typbar-TCV introduction. The primary outcome was monthly antimicrobial prescriptions in children aged 6 months to 15 years (vaccinated age group), normalised by the number of trauma events (including fractures, burns, road traffic accidents, and other accidental injuries or injuries resulting from assault; all age groups). Antimalarials, antivirals, anti-tuberculosis drugs, anti-helminthic drugs, and topical antibacterial agents were excluded. As a secondary outcome, we analysed the normalised rate of prescriptions of antimicrobials used for the treatment of typhoid (ciprofloxacin and azithromycin) in children.

Monthly counts of antimicrobials prescribed were normalised by the number of trauma events to control for secular changes due to incomplete recording and disruptions in health-care service provision. Trauma presentations were considered an appropriate control because they are unlikely to have been affected by the Typbar-TCV campaign.

To estimate vaccine effect on prescribing, a quasi-Poisson model was fitted to monthly prescription counts. We modelled the rate of prescriptions per trauma event by including monthly number of trauma events as an offset term in the model.^21^

Additionally, the model included: (1) an indicator for the post-vaccine period, (2) a linear term for time, and (3) sine and cosine terms to account for seasonality.^22^ Data analysis was done in R version 4.1.2 and made use of the nlme package.

Ethnographic data were entered into NVivo 14 for thematic analysis. An iterative approach was taken in which data were analysed alongside and fed back into the ongoing collection of data. Clinic survey and events mapping data allowed for high-level comparisons between the different clinics and the development of a provisional coding framework, which helped to refine directions and questions to be addressed during participant observation and in-depth interviews. Field notes, interview transcripts, survey responses, and workshop data were analysed using the coding framework, which was further refined in response to new themes and patterns as they emerged. Codes of progressively higher orders were developed and used to explain and theorise the findings across the different data sources. Regular meetings among the study team enabled the integration of qualitative ethnographic data and quantitative register data. The integration included, for example, use of the key events mapping to explain trends and gaps in registry data and use of ethnographic data to provide thick descriptions of the underlying complex decision-making processes. Register data provided more reliable estimates of antimicrobial prescribing, which helped to both mitigate recall bias and to refine the qualitative research questions. This iterative, bidirectional approach enabled us to derive our overall findings including a proposed conceptual framework for understanding the interaction between different factors and their effect on infections and antimicrobial use.

### Role of the funding source

The funder of the study had no role in study design, data collection, data analysis, data interpretation, or writing of the report.

## Results

Between Jan 1, 2018, and March 31, 2020, 62 211 clinic visits were recorded, of which 27 107 (43·6%) included children aged 6 months to 15 years. The median age was 2 years (IQR 1·2–5·0), 14 378 (53·0%) were male, and 12 729 (47·0%) were female (table 1). Almost two-thirds of child consultations (n=17 951; 66·2%) resulted in an antimicrobial prescription (appendix 2 p 5). Ciprofloxacin was prescribed for 2301 (8·3%) of 27 701 children, including 1612 courses (70·1%) prescribed for diarrhoeal disease and 208 for suspected typhoid (9·0%, appendix 2 p 5). Although ciprofloxacin was the main antimicrobial prescribed for diarrhoeal disease, amoxicillin was the most commonly prescribed antimicrobial, predominantly for respiratory illnesses (appendix 2 p 5). Respiratory tract infections were the most common diagnoses leading to antimicrobial prescribing in children. During this time, 3756 patients presented with trauma.

361 (1·3%) of 27 107 children were diagnosed with typhoid fever. The highest monthly number of suspected typhoid fever cases among children (n=85) was recorded in March, 2018, followed by two smaller peaks in October, 2018 (n=30), and February, 2019 (n=29). These peaks occurred during the rainy season in Zimbabwe.

Findings from the events mapping exercise (appendix 2 p 3) revealed that cholera, typhoid, and outbreaks of diarrhoeal disease were reported on an almost annual basis between 2016 and 2020, particularly during what health-care workers described as the typhoid and rainy season between October and February. Poor water sanitation and hygiene, combined with living in highly crowded areas, were highlighted as factors causing and contributing to disease outbreaks. With this context, health-care workers felt that vaccination contributed to a reduction in the number of patients presenting with typhoid symptoms (panel). Such perceived reductions, however, were rarely attributed to Typbar-TCV alone. Events mapping and survey data revealed several governmental and non-governmental organisation initiatives aimed at improving water and sanitation infrastructure, which were suggested to have had a role in reducing typhoid (panel). Health-care workers also highlighted COVID-19 lockdowns and the increased emphasis on hand hygiene as interruptions to typhoid transmission (panel). However, respondents stressed that poor water and sanitation infrastructure persists in many areas of the city; infrastructure deficiency, poverty, and overcrowding were thought to be drivers of ongoing diarrhoeal outbreaks and sporadic typhoid cases (panel). At the final collaborative workshop, some scepticism was expressed that typhoid had dropped as much as was apparent, citing reluctance to attend the clinic and self-prescribing practices as reasons why typhoid cases might not be identified.

**Figure 2:**
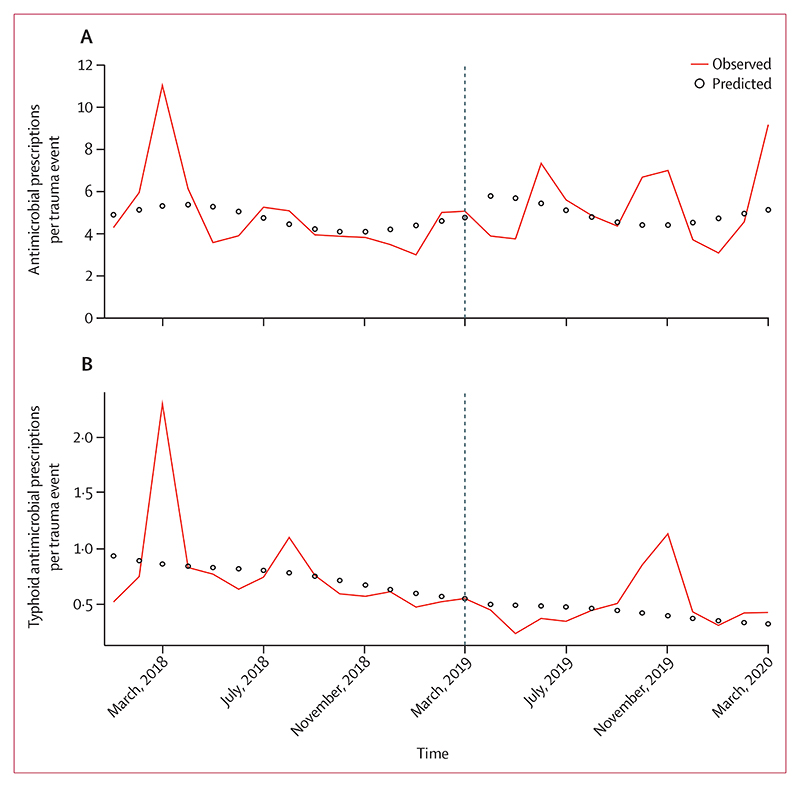
Observed monthly rates of prescriptions in children aged 6 months to 15 years (A) Prescription of any antimicrobials. (B) Prescription of antimicrobials recommended for typhoid fever. There was a 1% monthly decrease in prescriptions of any antimicrobials over the course of the study (adjusted rate ratio 0·99, 95% CI 0·96–1·04, p=0·62) and a 4% monthly decrease in antimicrobials for typhoid (0·96, 0·92–10·1, p=0·15).

The median monthly antimicrobial prescription rate was 4·28 (IQR 3·83–5·16) per trauma diagnosis in the pre-vaccination period and 4·70 (3·84–6·76) in the post-vaccination period. For typhoid antimicrobials median prescription rates were 0·65 (0·57–0·77) per trauma diagnosis in the pre-vaccination period and 0·44 (0·38–0·48) in the post-vaccination period (figure 2). Although a decreasing trend in antimicrobials commonly prescribed for typhoid was observed over time, the Typbar-TCV campaign had no effect on the rate of prescribing, either for all antimicrobials (adjusted rate ratio 1·20, 95% CI 0·70–2·05, p=0·505) or for typhoid antimicrobials (0·93, 0·44–1·96, p=0·85; figure 2, table 2). The same was true when restricting the analysis to prescriptions of typhoid antimicrobials in febrile children (appendix 2 p 6).

Qualitative data further explained why Typbar-TCV had not resulted in a demonstrable reduction in prescribing. Although several respondents believed, or at least hoped, that fewer typhoid-related antimicrobials had been used since the campaign, others noted that a combination of interventions would be needed to reduce prescribing (panel). As participant observation revealed, in practice, health-care workers found ruling out the possibility of typhoid infection challenging, meaning that they could not avoid prescribing antimicrobials. Notably, while the EDLIZ recommended laboratory confirmation of typhoid before antimicrobial treatment, such confirmation was rarely possible due to minimal access to diagnostics, specifically blood cultures. Respondents reported that transport of samples was infrequent and that results could take several days to come back. The reported cases identified in registers at the primary care clinics were therefore largely based on syndromic management of presumptive typhoid cases (panel).

The challenge health-care workers were then faced with was that the classic signs and symptoms of typhoid—fever, headache, and abdominal pain—were unspecific and could be caused by other infectious or non-infectious conditions. Several respondents explained the challenge of managing typhoid in this context of diagnostic uncertainty and the utility of antimicrobials in mitigating the infection (panel). Importantly, even when the Typbar-TCV vaccination status was known—and usually it was not—uncertainly about what could be causing the presentation meant that an antimicrobial might still be prescribed. Withholding an antimicrobial when the signs and symptoms were present could amount to discriminating against the patient (panel). Rates of antimicrobial prescribing identified in the clinic registers were also influenced by numerous macro-level and micro-level factors affecting service provision. These factors included staff shortages (due to outward migration), clinic closures, renovations, increased consultation fees, shortages of medicines, national hyperinflation, and industrial action (appendix 2 p 4). Although these stressors could account for decreased prescribing, they also exerted pressure to prescribe. The clinics were highly stressful environments for both patients and prescribers, and the time and resources for a thorough clinical examination to arrive at a probable diagnosis and to convey why an antimicrobial might not be needed were extremely limited.

As observed in consultations, when deciding whether to prescribe an antimicrobial, health-care workers took into consideration the socioeconomic vulnerability of their clients, which exposed them to pathogenic living conditions and compromised their ability to seek care. Cognisant of impoverished, unhygienic living conditions, the history of typhoid and other outbreaks, and knowing that this consultation might be the first and last time that they saw a particular patient, health-care workers often prescribed antimicrobials even if not recommended by the EDLIZ guidelines.

## Discussion

The mass typhoid conjugate vacinne (TCV) administration in Harare was the first time the new Typbar-TCV was administered through a mass vaccination campaign in Africa. This study found a very high rate of antimicrobial prescribing in children presenting to primary care clinics in Harare. Presentation with symptoms that might have been compatible with a typhoid-like illness were common. Despite the proven efficacy of Typbar-TCV and the observed reduction in typhoid cases in the vaccinated population,^9^ mass vaccination did not lead to a reduction in antimicrobial prescriptions in children presenting to primary care in Harare in the 12 months following the campaign.

This study shows the challenges resulting from the unavailability of diagnostic testing. Although the Typbar-TCV campaign led to a reduction in the number of typhoid cases, a trend observed by Harare City Health Authorities and front-line health-care workers, the difficulties in establishing diagnoses based on clinical symptoms, the persisting challenges of poor water and sanitation infrastructure, and awareness of patients’ social circumstances contributed to the fact that antimicrobials continued to be widely prescribed for typhoid and similarly presenting bacterial infections. There was a trend for reduced prescribing of antimicrobials that could be used to treat typhoid fever. However, the gradual decline was not affected by the Typbar-TCV campaign.

Studies with high efficacy or effectiveness of the new Typbar-TCV vaccine across different settings are encouraging given the low efficacy and durability of previous typhoid vaccines.^23^ Importantly the new Typbar-TCV vaccine was instrumental in curbing an outbreak of extensively drug-resistant *S* Typhi in Pakistan.^24^ An observational study in Harare estimated a 75% Typbar-TCV effectiveness in preventing culture-confirmed typhoid fever.^19^ A reduction in confirmed typhoid fever cases was also observed in children after the vaccination campaign.^9^ Following this 2019 campaign, TCV was introduced in the routine national immunisation programme in Zimbabwe in May, 2021.^17,25^

In addition to a direct reduction in disease burden, vaccines have been speculated to affect antimicrobial resistance by indirectly reducing antimicrobial prescribing.^13^ However, the magnitude of these effects is unclear and is probably related to disease epidemiology, prescribing patterns, and socioeconomic factors. Several studies focused on influenza,^11^ pneumococcal,^10,26^ rotavirus,^27^ and other childhood vaccines^28^ reported reductions in antimicrobial prescriptions following vaccination. However, most were done in high-income settings which generally have a lower burden of infectious diseases, better access to health care and diagnostics, and systems ensuring that patients can easily re-access care should they deteriorate clinically. A study using data from demographic health surveys done in low-income settings estimated that pneumococcal conjugate vaccines conferred 19·7% (95% CI 3·4–43·4) protection against antimicrobial-treated episodes of acute respiratory infection and live attenuated rotavirus vaccines conferred 11·4% (4·0–18·6) protection against diarrhoea.^29^ However, the reduction in antimicrobial use was assessed only in the 2 weeks preceding the interview and was based on caregiver report, which might lead to misclassification. Furthermore, the burden of pneumococcal and rotavirus infections in the population that can be prevented through vaccination is much higher than that of typhoid, which might explain the observed effect on antimicrobial use. Another study with a similar methodology from Nepal reported a reduction in antimicrobial use that was probably in conjunction with other measures.^28^

**Figure 3:**
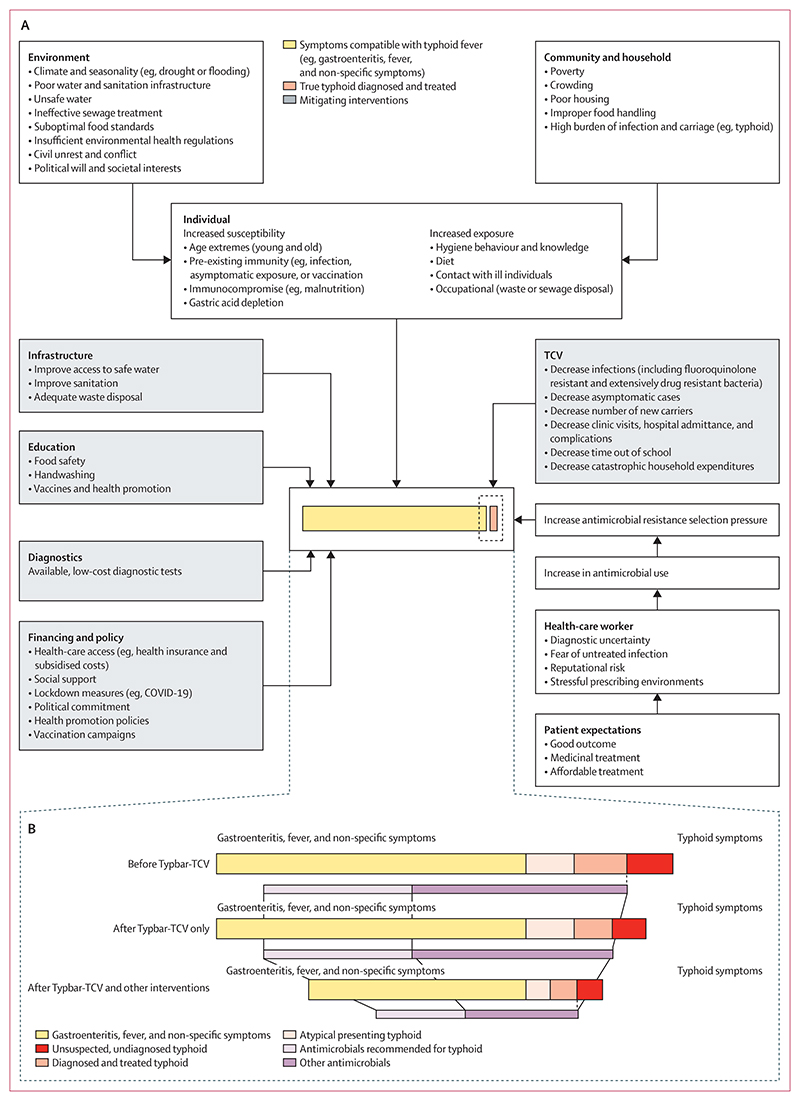
Conceptual framework of factors relating to typhoid fever, vaccination, and antimicrobial prescriptions (A) The interplay between drivers of typhoid fever and other infections, interventions that aimed to reduce the burden of infection, and antimicrobial resistance. (B) Hypothesised effect of Typbar-TCV and other system-wide interventions on antimicrobial use related to typhoid fever and other infections. TCV=typhoid conjugate vaccine.

In our study, a large proportion of children were prescribed antimicrobials; ciprofloxacin was frequently prescribed for acute gastroenteritis. Although Typbar-TCV is expected to substantially reduce typhoid burden in the community and, in turn, affect antimicrobial prescribing, our findings suggest the need to consider the effects of Typbar-TCV within a broader multidimensional framework, as emerged from our mixed methods analytic approach (figure 3). Typbar-TCV reduces the number of infections, with a substantial effect on transmission, morbidity and, potentially, household expenditures. However, the typhoid burden cannot be viewed in isolation from a large variety of complex environmental and socioeconomic factors that interact synergistically to cause a broad range of disease syndromes with considerable overlap in symptomatology, risk factors, and populations affected. Prescribing of antimicrobials for patients with suspected typhoid is inseparable from this broader scenario, as has been shown in a number of low-resource settings,^30,31^ including in Harare.^20^ In our study, despite health-care workers’ training and commitment to guideline adherence, in practice they were often unable to withhold antimicrobial treatment. However, considering that infection is challenging to diagnose, because of low availability of diagnostics, the non-specific symptoms patients present with, and underlying socioecological conditions that only slightly improved, this result is unsurprising.

Our findings suggest that for TCV to bring about sustainable reductions in prescribing antimicrobials, an integrated implementation strategy alongside other interventions sensitive to socioeconomic and health system contexts will be required. This strategy must include political will to sustain current efforts to redress the long-standing problem in this setting with water and sanitation infrastructure,^32^ efforts to improve availability of diagnostics and the supply of essential medicines, context-sensitive education and awareness initiatives, and strategies to improve access to health care and social protection mechanisms among groups who are socioeconomically disadvantaged. Scholarship across the health sciences has long highlighted a problematic tendency in global health programming to rely on technological, disease-specific solutions to entrenched health challenges at the expense of broader-based efforts to prevent diseases and strengthen health systems.^33,34^ In the context of antimicrobial resistance, this tendency has been responded to with increasing awareness of the need for antimicrobial resistance-sensitive interventions that account for the upstream drivers of infections, as well as antimicrobial resistance-specific interventions that more directly address the development of resistance.^35^ Our findings suggest that TCV is unlikely to reduce prescribing in a real-world low-income setting without also holistically responding to the conditions that make typhoid an archetypal disease of poverty.

Our study is limited by the use of routine records of antimicrobial prescriptions. Data from routine records might be incomplete due to staff shortages and high workload. As in many low-resource settings, electronic records, documenting presentations, and prescriptions are not available in primary care clinics in Zimbabwe. To account for missing data we normalised the rates of prescriptions using trauma diagnoses, as trauma events are unlikely to be influenced by seasonality and are relatively stable over time. Although antimicrobial dispensing is traditionally highly regulated in Zimbabwe and pharmacy sales without prescriptions were infrequent,^36^ some patients might also have accessed antimicrobials from informal sources without presenting at the clinic. Because of the COVID-19 pandemic, which led to a strict national lockdown, data were censored in March, 2020. The lockdown caused severe restrictions in movement for both patients and health-care workers and caused clinic closures where no outpatient consultations were done. A longer post-intervention period would have increased the power of the study. Similarly, sustained changes in antimicrobial prescribing practices might require longer periods of follow-up after vaccination. Furthermore, there was substantial month-to-month variation in antimicrobial prescribing, which made evaluation of the effect of Typbar-TCV difficult. In terms of qualitative data, recall bias might have affected the validity of findings as data collection commenced 1 year after the Typbar-TCV campaign. Social desirability bias might also have influenced behaviour (eg, during consultation observations) and responses (eg, on the potential effect of Typbar-TCV on prescribing).

The study also has several strengths. The study used routinely collected data from medical records for a large number of children presenting to outpatient clinics in a low-resource setting. Routine data are generally difficult to collect in low-income to middle-income countries because electronic patient record systems are not available, data might be missing due to staff shortages and high workload, and paper records might be lost over time. Additionally, we used a mixed methods approach and triangulation between different data sources to confirm and provide context to our findings. The qualitative research in particular enabled us to understand what actually mattered to frontline prescribers and local actors and facilitated the production of an expanded framework for conceptualising the effects of Typbar-TCV and improved implementation strategies.

In Harare, the Typbar-TCV was highly effective in reducing typhoid fever cases.^9,19^ Overall antimicrobial prescriptions were very common in children with symptoms compatible with typhoid fever. We were not able to observe a change in antimicrobial prescriptions within 1 year following the Typbar-TCV mass campaign in 2019. This absence of observed effect could be explained by the context in which typhoid occurs. Typhoid is a human-restricted faecal–oral infection found in areas with poor water quality and suboptimal water and sanitation infrastructure. The same living conditions that put people at risk of typhoid disease also increase the risk of other diarrhoeal diseases. Antimicrobial prescribing practices are not just influenced by disease incidence, but by the health system itself, including access to diagnostics and essential medicines. Therefore, a multipronged package of interventions including vaccination, health system strengthening, and improved water sanitation and hygiene infrastructure is needed to affect antimicrobial use.

## Supplementary Material

Supplement 1

Supplement 2

## Figures and Tables

**Table 1:** Characteristics of clinic visits for children aged 6 months to 15 years according to vaccination period

	Pre-vaccination period (n=17 169)	Post-vaccination period (n=9938)
Female sex	8043 (46·8%)	4686 (47·2%)
Age (median [IQR])	2 (1·2–6·0)	2 (1·1–5·0)
6–23 months	6745 (39·3%)	4035 (40·6%)
2–4 years	4665 (27·2%)	3250 (32·7%)
5–9 years	4281 (24·9%)	2075 (20·9%)
10–15 years	1478 (8·6%)	578 (5·8%)
Febrile on presentation	3768 (28·8%)	1297 (22·8%)
Diagnosed with clinical typhoid	266 (1·5%)	95 (1·0%)
Treatment prescribed		
Any treatment	15 095 (88·9%)	8568 (87·7%)
Any antimicrobial	11 908 (69·4%)	6043 (60·8%)
Ciprofloxacin	1858 (10·8%)	443 (4·5%)
Azithromycin	39 (0·2%)	96 (1·0%)
Referred to hospital	2254 (13·3%)	1112 (11·4%)

Data are n (%) unless otherwise specified. 8325 patients were missing a temperature measurement, 353 were missing information on treatment, and 360 on hospital referral.

**Table 2:** Multivariable quasi-Poisson regression analysis for all antimicrobial prescriptions and typhoid antimicrobial prescriptions in children aged 6 months to 15 years

	Pre-vaccination (n=17 169)	Post-vaccination (n=9938)	Adjusted rate ratio	p value
All antimicrobial prescriptions	11 908 (69·4%), 4·62	6043 (60·8%), 5·12	1·20 (0·70−2·05)	0·51
Typhoid antimicrobials (ie, ciprofloxacin and azithromycin)	1895 (11·0%), 0·74	539 (5·4%), 0·46	0·93 (0·44−1·96)	0·85
Trauma presentations	2576 (15·0%), ref	1180 (11·9%), ref	1 (ref)	··

Data are n (%), rate per trauma cause; adjusted rate ratio (95% CI); or p value. Trauma included fractures, burns, road traffic accidents, and other accidental injuries or injuries resulting from assault. Adjusted rate ratio from quasi-Poisson regression model adjusted for trend and seasonality.

## Research in context

### Evidence before this study

Antimicrobial resistance is a considerable threat to human health. One of the main drivers of antimicrobial resistance is antimicrobial use. Vaccination has been proposed as a possible tool to mitigate antimicrobial resistance by reducing the overall infection burden, including the burden of infections due to antimicrobial resistant organisms, subsequently decreasing antimicrobial need. A 2019 systematic review explored the effect of vaccination on antimicrobial use; most identified studies were in high-income settings; this previous Article reviewed the available evidence from studies published berween 1998 and 2018 and we did an updated MEDLINE search to identify studies describing the effect of vaccination on antimicrobial prescribing and use in low-income and middle-income settings. The MEDLINE search was conducted for articles published between Jan 1, 2018, and Dec 5, 2022, using the terms “vaccination” or “immunisation” AND “antibiotic” or “antimicrobial” AND “prescriptions” or “use”, and related terms. Studies using mathematical modelling, and those restricted to patient subgroups (eg, patients admitted to hospital with pneumonia) were excluded. The search identified 324 publications of which 27 were reviewed in full text and three were relevant to our study. Two studies, based on household surveys assessing illness and antimicrobial use in the 2 weeks preceding the survey, reported reductions in antimicrobial use for respiratory infections as a result of routine childhood vaccines. The third study used aggregated vaccine and antimicrobial consumption data from the private health-care sector in India. This study reported an annual increase in vaccine and antimicrobial use, but a reduction in antimicrobial consumption with increased vaccination when using monthly data and an extended lag time of almost 3 years.

### Added value of this study

Our study reports the findings from 27 107 child outpatient consultations done before and after a mass vaccination campaign using a new typhoid conjugate vaccine in Harare, Zimbabwe. Although a decreasing trend was observed for antimicrobials commonly prescribed for typhoid fever, the typhoid conjugate vaccine campaign did not affect antimicrobial prescribing in this setting. Qualitative data also supported these findings. Health-care workers believed that there had been a reduction in typhoid cases, in part due to the typhoid vaccine. However, diagnostic uncertainty and recognition of the socioeconomic circumstances of parents and children—including poor water and sanitation infrastructure and the challenges of accessing care were the child to deteriorate—continued to favour overprescribing of antimicrobials. To our knowledge, this study is the first to use routine outpatient consultation data from a low-resource setting to evaluate whether vaccine use can affect antimicrobial prescribing.

### Implications of all the available evidence

This study highlights many of the challenges faced by health-care workers in low-resource settings in relation to antimicrobial prescribing (eg, diagnostic difficulties leading to uncertainty, patient vulnerability, and challenges in accessing health care), which can explain the absence of an observable effect. More comprehensive strategies integrating health systems strengthening, improved water sanitation and hygiene, access to diagnostics, and vaccination are needed to decrease antimicrobial prescribing in low-resource settings.

## *Panel*: Interview and workshop excerpts

### Perceptions of the effect of Typbar-TCV on typhoid cases

- “Yes, it [typhoid] decreased remarkably…For example, like I said [suburb name] was our hotspot for typhoid but then, after the vaccination, we felt that the numbers went down.” (Nurse, female aged 41 years, non-vaccination clinic)
- “The vaccine must logically have played a role in reducing typhoid.” (Harare City Health official, collaborative workshop)

### Factors continuing to drive diarrhoeal outbreaks and sporadic typhoid cases

- “You will realise that those people continue to want to use water from the wells… last year [2021] in April or May… I remember there was about 15 [typhoid] cases…after these vaccination campaigns had taken place… the water was contaminated because there is a sewerage line that is there.” (Environmental health technician, male aged 56 years, vaccination clinic)
- “High density communities, with 3–5 families living in one house…without proper garbage collection and accumulating dirt… and water that is not that clean or suitable for drinking…” (Nurse, male aged 43 years, vaccination clinic)

### Perceptions of effect of Typbar-TCV on antimicrobial prescribing

- “The prescribing of antimicrobials especially to under 5’s has decreased because of the TCV, which has caused a great decrease in typhoid.” (Nurse, female aged 38 years, vaccination clinic)
- “Yes there is a difference, I don’t hear or see anyone getting ciprofloxacin prescription, mainly because there are no diarrhoea cases these days.” (Nurse, female aged 54 years, vaccination clinic)
- “I would like to think the vaccine made a difference. When I was in [clinic name], we had many cases and after the vaccine there were significantly less cases. Now there are no typhoid suspects and therefore we do not prescribe antimicrobials, as per guidelines.” (Nurse, female aged 52 years, non-vaccination clinic)
- “We need a combination strategy, no single intervention [TCV] will reduce prescribing.” (Harare City Health official, collaborative workshop)

### Reasons for prescribing without laboratory confirmation of infection

- “We would really, really love to give an antimicrobial that’s based on lab culture results but the fact we don’t have an on-site lab, it might take you know a couple days before we get a result and so the drug of choice that we are, we were using was ciprofloxacin.” (Nurse, male aged 43 years, vaccination clinic)

### Challenges of diagnosing typhoid when symptoms overlap with other conditions

- “The challenge that we face is, it [typhoid] can be confused for other conditions, for example, acute abdominal pain for someone with a ruptured appendix, it can be similar to typhoid, and then people who have indigestion can be confused to be having typhoid when they actually do not have it.” (Nurse, female aged 41 years, non-vaccination clinic)
- “Right now we are in the time of COVID…so any person with a high temperature it’s assumed to be COVID-19…because we give an antimicrobial, then all ailments you have will disappear whether it was typhoid, or whether it was COVID… so I am saying you will get better anyway even if you were missed out and not recorded as typhoid…” (Nurse, female aged 58 years, vaccination clinic)

### Hesitancy to withhold antimicrobials, even among vaccinated patients, due to diagnostic uncertainty

- “For now, I think I would [prescribe antimicrobials] because if someone is already sick, there is no need for me to discriminate them and say because they were vaccinated they should be able to fight it. I will prescribe what I will prescribe for someone who hasn’t had the vaccine.” (Nurse, female aged 41 years, non-vaccination clinic)

## Data sharing

The data supporting the findings of this study are available within the Article and its supplementary materials. Individual de-identified participant data, data dictionaries, and the study protocol will be made available on Dryad (www.datadryad.org) within 12 months of publication. Data will be shared with researchers upon reasonable request to the corresponding author.
