# Supplementary material for: The effect of a comprehensive typhoid conjugate vaccine campaign on antimicrobial prescribing in children in Harare, Zimbabwe: a mixed methods study
Source: Lancet Glob Health. Author manuscript; Available in PMC 2024 Jun 7. (PMC7616073; doi:10.1016/S2214-109X(23)00319-4)
Supplement: Supplement 1 [file EMS196653-supplement-Supplement_1.pdf]

THE LANCET
Global Health

Supplementary appendix 1

This translation in Shona was submitted by the authors and we reproduce it as supplied. It has not been peer reviewed. *The Lancet*'s editorial processes have only been applied to the original in English, which should serve as reference for this manuscript.

Shanduro iyi muShona yakatumirwa nevanyori uye tinoiburitsa sekupihwa kwatakaitwa. Huye hayina kuongororwa nevezera. Maitiro ekupepeta e Lancet angoshandiswa chete kugwaro rekutanga, iro rinofanira kushanda seumboo hwechinyorwa.

Supplement to: Olaru ID, Chingono RMS, Bottomley C, et al.
The effect of a comprehensive typhoid conjugate vaccine campaign on antimicrobial prescribing in children in Harare, Zimbabwe: a mixed methods study. *Lancet Glob Health* 2023; **11:** e1422–31.

Background Ma **vaccine** anodzivirira zvirwere uye anoderedza kushanda kwemishonga ye***antimicrobial***. Kubaiwa kwenhomba ye*Typbar-TCV* (Bharat Biotech, India) kune veruzhinji kwakaitwa kwesvondo imwechete kubva musi wa 25 Kukadzi kusvika musi wa 4 Kubvumi, mugore ra2019. Takaongorora kuti tizive kana mushonga uyu wekudzivirira utachiona *hwetyphoid conjugate* uchakonzera shanduko mukunyorwa kwemishonga we *antimicrobial* kune vana vadiki vari kuonekwa muzvipatara zvemuHarare, Zimbabwe.

Methods Mutsvakurudzo ino yakaitwa kuchishandiswa nzira dzakasangana, humbovo hwekuongororwa kwevechidiki panzvimbo dzavanosvikira kuma kiriniki hwakatorwa kubva musi wa Jan 01, 2018, kusvika March 31 2020, kumakiriniki mashanu emu Harare. Ongororo ye *time series analysis* yakaitwa kuenzanisa humbovo hwemishonga yakapihwa pasati pave nekurudziro (*campaign*) uye mushure mekurudziro. Kuti zvakawanikwa mutsvakurudzo zvitsanangurwe, humbovo hwakaunganidzwa kubva muna Kubvumbi 20, 2021, kusvika munaChikunguru 20, 2022 zvichisanganisira zvakabva muongororo ye *ethnography* (kureva, ma*workshop*, ma*survey* , ma*observations*, uye ma *interviews*) zvaibva kumakiriniki gumi nemana. Zvakabuda kubva paongororo dze *ethnography* zvakashandiswa kuita ongororo dze *thematic analysis*. Chikuru chaitarisirwa pakupera kwetsvakurudzo kuziva mishonga we*antimicrobial* pamwedzi woga woga wakanyorerwa vana vechidiki vane mazera anotangira pamwedzi mitanhatu yekuzvarwa kusvika pamakore makumi mashanu, takatarisa nhamba yezviitiko zvaishungurudza kumapoka emazera ose.

Findings Panguva yakaunganidzwa humbovo muongororo, pakawanikwa humbovo kubva kune vechidiki vanosvika 27 107. Pane vechidiki 27 107 ava chikamu chinosvika 17 951 (66·2%) vakanyorerwa mishonga ye *antimicrobials*. Zvisinei nefungidziro iripo yekudzikira kwevari kuwanikwa vaine *typhoid,* uye kuderera kwekunyorwa kwemishonga yema *antimicrobials* inowanzo shandiswa pakurapa *typhoid* (yakaita se, ciprofloxacin ne azithromycin) kubaiwa kwenhomba ye*Typbar-TCV* kune ruzhinji hakuna kushandura huwandu hwema *antimicrobial*s (adjusted rate ratio, 1·20, 95% CI 0·70–2·05, p=0·51) kana huwandu hwemishonga *ye*ma *antimicrobials* yainyorerwa kurapa *typhoid* (0·93, 0·44–1·96, p=0·85) pane vose vakaonekwa. Zvitubu zvemvura zvisina kuchengetedzeka uye kushomeka kwenzira dzinoshandiswa pakuvheneka nepakuongorora utano zvirikubatsiridza mukuenderera mberi kwechirwere uye kunyorwa/kupihwa kwemishonga yema*antimicrobial* inorwisa mabhakitiriya.

Interpretation Hurwere hwe*febrile* (zviratidzo zvekupisa muviri) hunokonzerwa ne *typhoid* yabatwa mumiviri kana irikufungidzirwa iri mumuviri ndihwo hunonyanya konzera kushandiswa kwemishonga yema*antimicrobial* inorwisa mabhakitiriya munzvimbo dzine dambudziko. Kunyange zvazvo yakashanda mukudzivirira *typhoid fever*, hatina kukwanisa kuziva mugumo wekushanda kwe*Typbar-TCV* pakunyorerwa/kupihwa kwema*antimicrobial (*mishonga yekudzivirira bhakitiriya). Tsvagiridzo ye*ethnography* yakaratidza maonero evazhinji takanangana nevatariri vemishonga yema*antimicrobial*, kusanganisira zvinetswa panyaya yekuwana mvura yakachena, kuraswa kwetsvina yemasuwa nenzira dzakakodzera, mabatirwo panyaya dzezvehutano uye kuvepo kwezvinovheneka kana kuongorora hutano. Kuti tione shanduko kunopfuura kuderedza mutoro wechirwere, panodiwa kubatanidza nzira dzakawanda dzekugadzirisa matambudziko aya kuitira kuti kukosha kwejekiseni renhomba (*vaccines*) pakuderedza kushandiswa kwemishonga yema*antimicrobial* kuonekwe zvakazara.
