# Supplementary material for: The effect of a comprehensive typhoid conjugate vaccine campaign on antimicrobial prescribing in children in Harare, Zimbabwe: a mixed methods study
Source: Lancet Glob Health. Author manuscript; Available in PMC 2024 Jun 7. (PMC7616073; doi:10.1016/S2214-109X(23)00319-4)
Supplement: Supplement 2 [file EMS196653-supplement-Supplement_2.pdf]

THE LANCET
Global Health

Supplementary appendix 2

This appendix formed part of the original submission and has been peer reviewed. We post it as supplied by the authors.

Supplement to: Olaru ID, Chingono RMS, Bottomley C, et al.
The effect of a comprehensive typhoid conjugate vaccine campaign on antimicrobial prescribing in children in Harare, Zimbabwe: a mixed methods study. *Lancet Glob Health* 2023; **11:** e1422–31.

Supplementary materials

Title: Can a comprehensive typhoid conjugate vaccine campaign impact antimicrobial prescribing in children living in Harare, Zimbabwe: a mixed methods study

Table of Contents

Fig S1. Geographical location of clinics 2

Setting 2

Data collection and management 2

Fig S2. Calendar of events used to understand factors that may have impacted on clinic presentations and antimicrobial prescribing 4

Fig S3. Antimicrobial prescriptions in primary care in Harare. 5

Fig S4. Observed monthly rates of prescriptions for typhoid antimicrobials in febrile children 6

Fig S5. Observed monthly rates of typhoid diagnoses in children 6

Table S1. Regression coefficients for the two analyses 7

Table S2. Qualitative methods breakdown 7

Fig S1. Geographical location of clinics

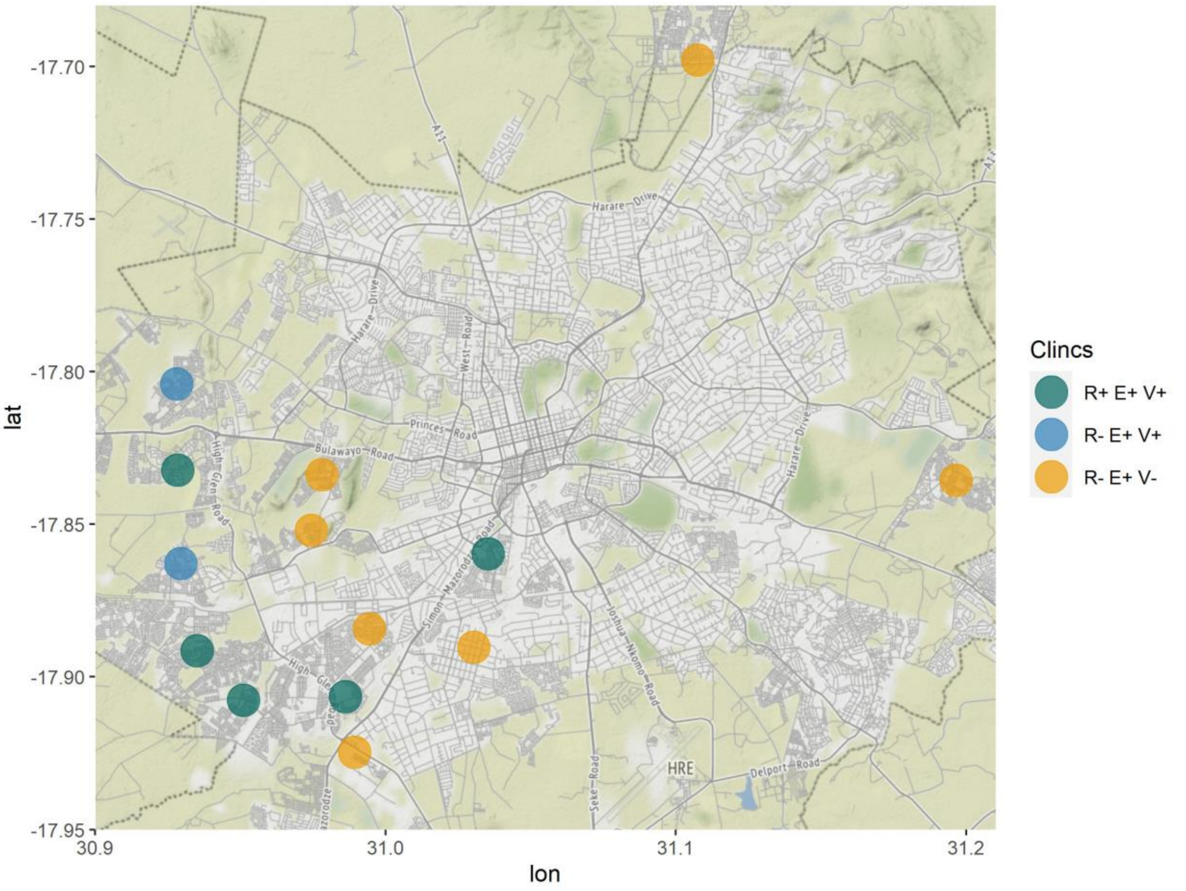

Green: clinics with register (R+) and ethnographic (E+) data available from suburbs where vaccination was conducted (V+); blue: clinics with ethnographic (E+) data from suburbs with vaccination (V+); orange: clinics with ethnographic (E+) data from suburbs without vaccination (V-).

Setting

All clinics included in this study provided primary care to patients residing in the respective suburbs. Primary care clinics represent the first port of call for patients (prior to presentation to higher-level facilities). These clinics provide outpatient consultations for acute illnesses, antenatal care and maternity services for uncomplicated deliveries, well-child and immunisation services, and HIV and tuberculosis services. Severe cases are referred to hospital. The routine data was collected from records at the primary care clinics. All clinics in this study were located in high-density suburbs and served a low-income population. High-income suburbs were not affected by the typhoid outbreak and were not included in the TCV campaign (or in the study).

The TCV mass campaign was conducted between 25 February and 4 March 2019 in nine suburbs in Harare. Overall administrative vaccination coverage was high of 72% for children aged 6 months to 4 years and 97% for those aged 5 to 15 years. Among the clinics included in our study vaccination coverage (administrative) was reported as follows: Budiriro 79·6%, Glen Norah 100·7%, Glen View 88·2%, Kuwadzana 90·7%, Mbare 79·5%.[1] Ciprofloxacin and azithromycin are antimicrobials commonly used in outpatients with suspected typhoid fever in accordance with the Zimbabwean National guidelines.[2]

Data collection and management

Data collection: In preparation for this study, the most common diagnoses and medicines prescribed were identified and coded in the electronic data collection form (entered from a list of options). If the diagnoses/ medicines deviated from the respective list, they were typed as free text by the research staff. Medicines were almost exclusively entered from the list of options (as a limited number of medicines are available in the clinic

pharmacies). Thus medicines usually did not require any further recoding. All critical variables were designed as "must enter" for the electronic data collection forms to minimise missing data. Because of the nature of the routine medical records (line list of patient presentations), the number of variables was limited. This included: clinic, date of presentation, age, sex, medicines prescribed, diagnosis, referral to hospital.

Data cleaning: Diagnoses and medicines that were not assigned from the list, were recoded based on letter combinations and manual coding (by tabulating presentations that were not assigned to a diagnosis and assigning them). Recoded diagnoses were manually checked for accuracy. Coding was done by a medical doctor.

Fig S2. Calendar of events used to understand factors that may have impacted on clinic presentations and antimicrobial prescribing

TAZ

Impact of a Typhoid Conjugate Vaccine Campaign on Antimicrobial Prescribing in Harare, Zimbabwe (TAZ)

2019

January	February	March	April
Cholera and typhoid outbreak.<br><br>Ciprofloxacin resistant typhoid in clinic 1	TCV awareness (2nd February)<br><br>MSF typhoid screening in Clinics 1, 2, 3<br><br>TCV roll out nationwide (25 February- 04 March)	Zero fees directive for diarrheal symptoms<br><br>Oral cholera vaccine campaign	
**May**	**June**	**July**	**August**
		Clinic 4 was established and has never closed	Yearly user fees review
**September**	**October**	**November**	**December**
	Industrial action by nurses e.g., in clinic 5	Industrial action by nurses e.g., in clinic 5<br><br>Decreased attendance due to skeleton staff	Industrial action by nurses

TAZ

Impact of a Typhoid Conjugate Vaccine Campaign on Antimicrobial Prescribing in Harare, Zimbabwe (TAZ)

2020

January	February	March	April
Countrywide medicines stockouts (Jan to Dec)		COVID-19 outbreak and national lockdown<br>Medicine stockouts countrywide<br>Clinic 7 closed for 10 days due to COVID-19 clients went to hospital 1	Attendance decreased due to the lockdown until December<br><br>Medicine stockouts countrywide.
**May**	**June**	**July**	**August**
	Attendance decreased due to the lockdown<br><br>Clinic 8 PPE stockout	Industrial action/ Nurses strike until December<br>Quarantine of staff in COVID-19 affected facilities<br>Staff shortage<br>Closure of 9 clinics until December<br>Clinic 8 PPE stockout	Clinic 9 closure clients went to clinic 2<br><br>Clinic 5 closure, clients went to clinic 1<br><br>Clinic 2 PPE shortage
**September**	**October**	**November**	**December**
Clinic 9 closure<br><br>Clinic 5 closure<br><br>Clinic 2 PPE shortage	Clinic 9 closure<br><br>Clinic 5 closure<br><br>Clinic 2 PPE shortage	Clinic 9 reopens<br><br>Clinic 5 reopens	Industrial action<br><br>Staff shortage<br><br>Closure of 9 clinics from July

MSF: Médecins Sans Frontières; PPE: personal protective equipment; TCV: typhoid conjugate vaccine.

Fig S3. Antimicrobial prescriptions in primary care in Harare. A: Prescriptions of different antimicrobials according to diagnosis in children; B: Prescriptions of antimicrobials recommended for typhoid according to diagnosis in children.

A

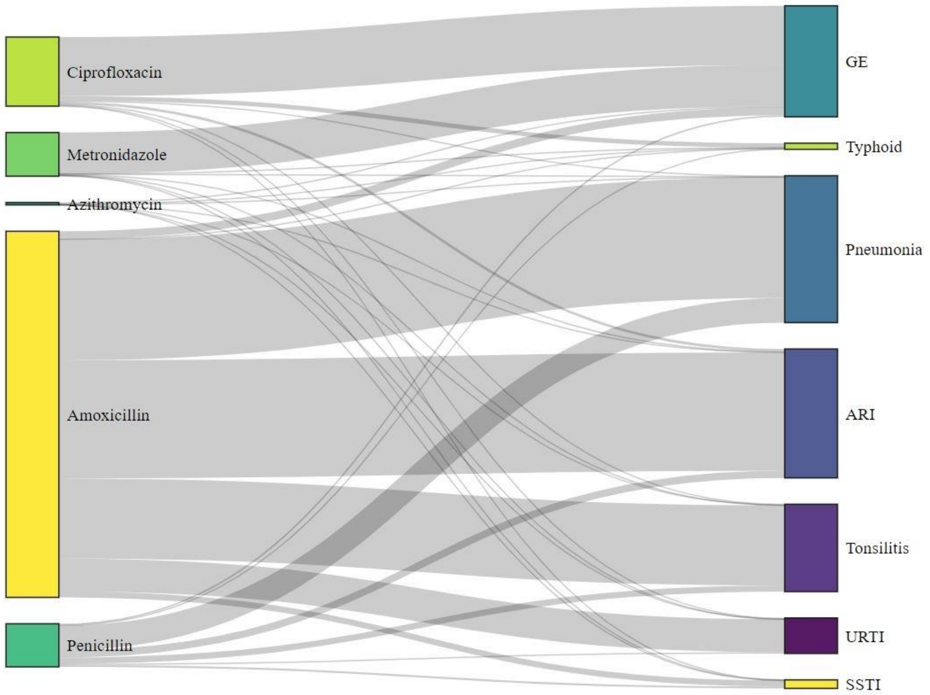

B

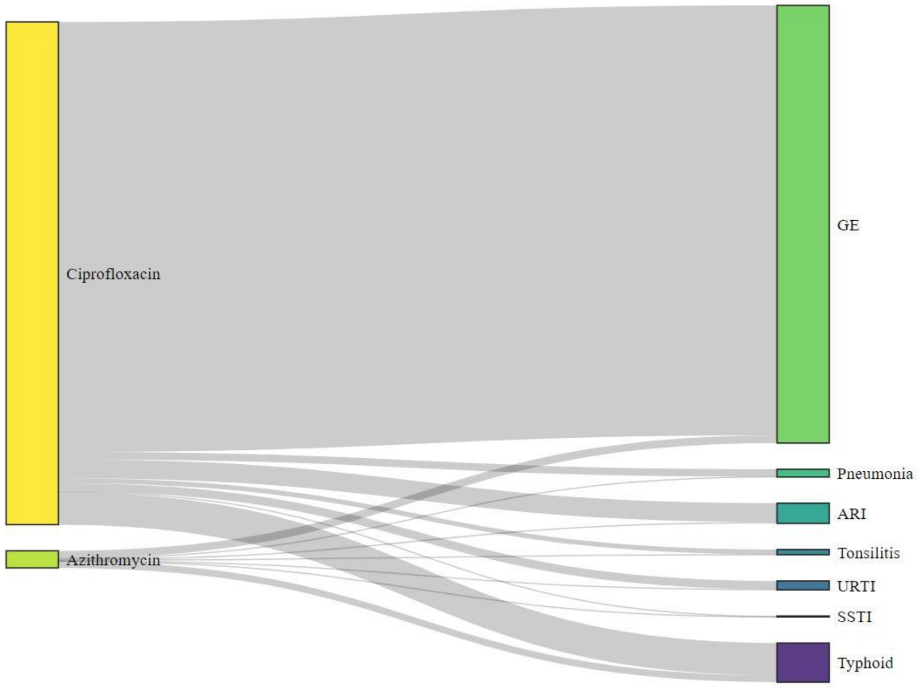

ARI: acute respiratory infection; GE: gastroenteritis; SSTI: skin and soft tissue infection; URTI: upper respiratory tract infection. 80% of patients who did not receive antimicrobials for typhoid fever were referred to hospital for treatment.

Fig S4. Observed monthly rates of prescriptions for typhoid antimicrobials in febrile children aged 6 months to 15 years (red). Hollow circles show predicted rates according to the model

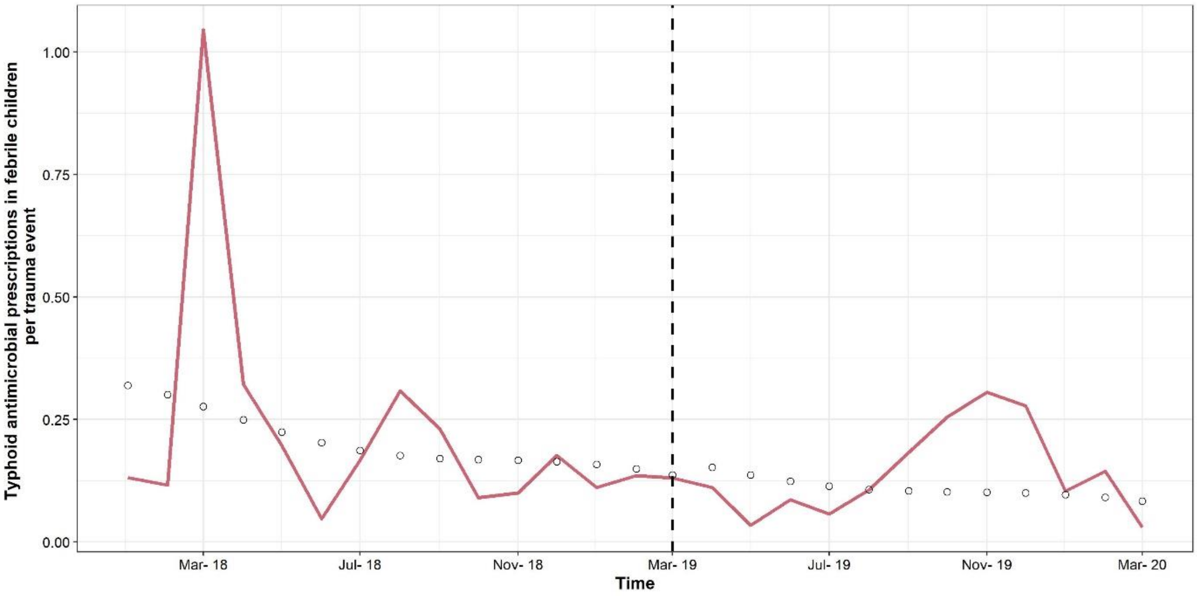

Rates are normalised using trauma diagnoses.

Fig S5. Observed monthly rates of typhoid diagnoses in children aged 6 months to 15 years (black line). Hollow circles show predicted rates

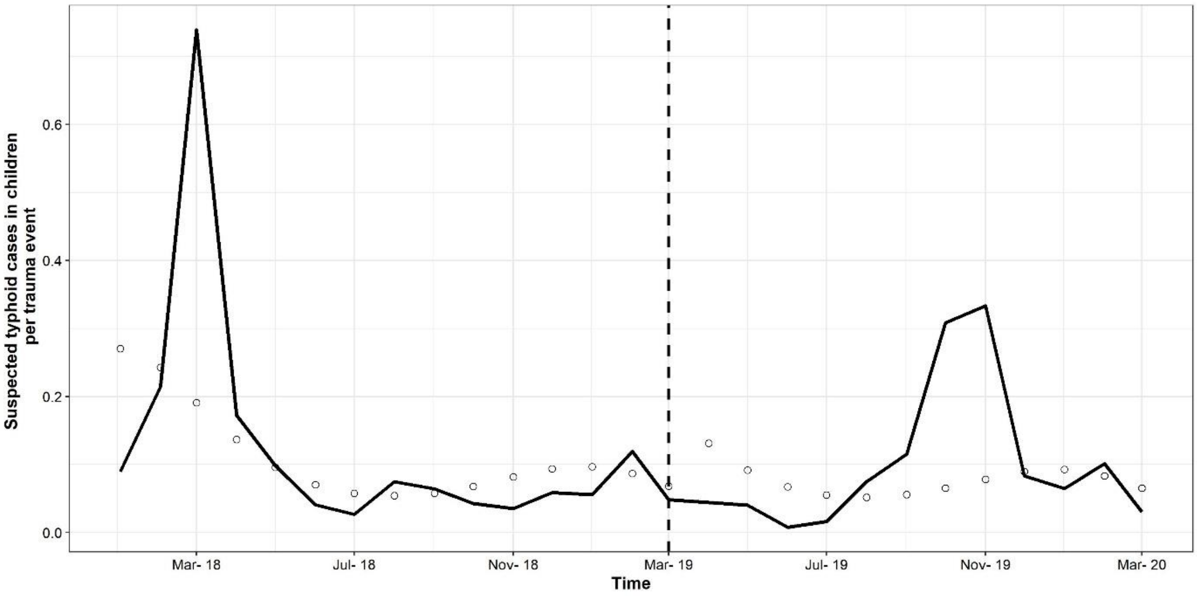

Table S1. Regression coefficients for the two analyses

Variable	Change in antimicrobial prescribing (log rate, SE)	p-value	Change in typhoid antimicrobial prescribing (log rate, SE)	p-value
[Intercept]	1·61 (0·18)	<0·001	0·013 (0·22)	0·956
TCV effect	0·184 (0·27)	0·505	-0·074 (0·38)	0·849
Time in months	-0·009 (0·02)	0·622	-0·036 (0·02)	0·150
Seasonality (sine, cosine)	0·089 (0·10)<br>-0·065 (0·11)	0·364<br>0·574	-0·042 (0·13)<br>-0·002 (0·15)	0·751<br>0·910

TCV: typhoid conjugate vaccine; SE: standard error

Table S2. Qualitative methods breakdown

Method	Description	Participant groups	Researcher(s)	Dates
Key events mapping workshop	Aim: to contextualize the occurrence of typhoid within the Harare socio-economic context by mapping key events that occurred between 2016-2021, that bear both directly and indirectly on typhoid and its management.<br><br>How: A participatory workshop guide was developed with questions trying to characterise they typhoid burden in Zimbabwe, its pattern of occurrence, associated risk factors at household level, health facility level, community level, and national level. Participants worked on an interactive calendar (Fig S2) to collaboratively map out the events. The duration of the workshop was 3 hours. Detailed workshop notes were taken by the research assistants, and later written up. After the workshop, follow-ups were conducted with 6 matrons to further fill in details that could not be recalled during the workshop and required identifying relevant documents	Harare City Health officials (n=19) responsible for preventative and curative primary care within the City districts.	Postdoctoral researcher (RMSC) and two research assistants (FRK and CAN)	June 2021
Clinic survey	Aim: to capture community-specific events not discernible at the Harare City-level to gain a formative understanding of clinic routines and typhoid management.<br><br>How: The survey tool was developed and included questions asking about staffing and services, major events affecting the clinic opening hours/services, typhoid management, any changes since the TCV (or since the last survey), antibiotic stocks, and resource challenges. They were conducted in 14 health facilities at two timepoints, once at the start and once at the end of data collection.<br>The surveys went through a preliminary analysis, and the findings were used to sample a smaller number of clinics in which to focus participant-observation and interviews. The initial survey findings, and other findings from the study, were also used to refine questions for the follow-up survey conducted in May 2022.<br>The survey was administered using Open Data Kit (ODK) on android tablets. The data was uploaded daily onto a London School of Hygiene and Tropical Medicine secure cloud storage.	14 facility nurse in-charge or acting nurse in-charge	Two research assistants (FRK and CAN)	June 2021 and May 2022
Participant-observation	Aim: to gain a granular understanding of typhoid prevention, diagnosis and treatment, the factors taken into consideration (including vaccination status), and any challenges of following the treatment guidelines.<br><br>How: it was conducted in a sub-set of 8 purposively selected clinics informed by findings from the clinics survey (e.g., to represent vaccination and non-vaccination clinics, experiences of typhoid outbreaks, known challenges of water and sanitation, etc.).<br>Research assistants (RAs) worked in each clinic for approximately 2 - 4 weeks (depending on the size of the clinic), writing detailed field notes to capture both general clinic routines and specifically spending time observing prescribing practices in the clinic outpatient departments (where typhoid cases would be seen). In the consultation rooms, the RAs observed how cases were managed, observations and tests performed, diagnoses made, medicines prescribed, and where patients were referred from the	26 Outpatient department nurses (n=18), and environmental health technicians (n-8)	Two research assistants (FRK and CAN)	October 2021 – May 2022

	consultation rooms. The RAs paid attention to the social dynamics between the nurses and the patient and the resource constraints shaping the interaction. The RAs also went out into community settings with environmental health technicians to better understand the social setting, the WASH and typhoid situation in the communities served by the clinics. Detailed observation fieldnotes were taken.			
In-depth interviews	Aim: To probe in further detail about events observed during participant observation, and to gain a broader understanding of typhoid, prescribing and TCV from a range of stakeholders.<br><br>How: A semi-structured topic guide was developed with questions that probed further about social phenomena observed during participant-observation (often there was not enough time, or it was inappropriate for in-depth questions in the consultation rooms), The other questions were on the perceptions about typhoid and the TCV, about typhoid management and antimicrobial prescribing, and general challenges of healthcare provision. In-depth interviews were conducted with a range of healthcare workers and stakeholders including clinic nurses, clinic pharmacy dispensary attendants, environmental health technicians and facility managers (n=22).<br><br>Interviews were conducted, in English or if preferred the local language (Shona), and they were conducted in a private space at a time/place that was acceptable to the participant – usually an empty room at the clinic for healthcare workers/facility managers. They were between 30-60 minutes long. Interviews were audio-recorded on an encrypted device and transcribed and translated into English.	All participant groups (n=22)	Postdoctoral researcher (RMSC) and two research assistants (FRK and CAN)	June 2021, and between October 2021-June 2022
Collaborative dissemination workshop	A final collaborative dissemination workshop was held with Harare City Health authorities. The aim of the workshop was to relate our findings to the assembled stakeholders, to collaboratively interpret and drawing out their significance, and to work towards some key recommendations for reporting back to the Ministry of Health and Child Care.	Harare City Health Authorities (n=28)	RMSC, KK, JD, FRK, CAN	April 2023

1. Poncin M, Marembo J, Chitando P, et al. Implementation of an outbreak response vaccination campaign with typhoid conjugate vaccine - Harare, Zimbabwe, 2019. *Vaccine X* 2022; **12**: 100201.

2. EDLIZ 2015: 7th Essential Medicines List and Standard Treatment Guidelines for Zimbabwe: National Medicine and Therapeutics Policy Advisory Committee; 2015.
